# RISK6  as a Translational Host Transcriptomic Signature for Tuberculosis Diagnosis, Treatment Monitoring, and Risk Stratification  

**DOI:** 10.3390/pathogens15050489

**Published:** 2026-05-01

**Authors:** Chrispian Mamudi, Purnamawati, Panca Andana, Prayudi Santoso, Lidya Chaidir, Arto Soeroto

**Affiliations:** 1 Department of Doctoral Program in Medical Science, Faculty of Medicine, Padjadjaran University, Bandung 40161, Indonesia; 2 Division of Respirology and Critical Care, Department of Internal Medicine, Faculty of Medicine, Krida Wacana Christian University, Jakarta 11510, Indonesia; 3 Department of Histopathology, Faculty of Medicine, Krida Wacana Christian University, Jakarta 11510, Indonesia; purnamawati@ukrida.ac.id; 4 Department of Biomedical Sciences, Faculty of Medicine, Padjadjaran University, Bandung 40161, Indonesia; pancaa99@gmail.com (P.A.); lidya.chaidir@unpad.ac.id (L.C.); 5 Division of Respirology and Critical Care Medicine, Department of Internal Medicine, Faculty of Medicine, Padjadjaran University, Bandung 40161, Indonesia; prayudi@unpad.ac.id (P.S.); a.y.soeroto@unpad.ac.id (A.S.)

**Keywords:** tuberculosis, RISK6, host-response biomarkers, transcriptomic signature, diagnosis, treatment monitoring, disease progression, multidrug-resistant tuberculosis

## Abstract

Tuberculosis (TB) remains a leading cause of infectious mortality worldwide, reflecting persistent gaps in diagnosis, risk stratification, and treatment monitoring. Host RNA transcriptomic signatures have emerged as promising tools for capturing dynamic immune responses across the TB disease spectrum. Among these, the six-gene RISK6 signature has attracted attention due to its parsimonious design and potential for clinical translation. This review provides a clinically oriented synthesis of current evidence on host transcriptomic biomarkers, with a particular focus on the application of RISK6 in diagnosis, prediction of disease progression, and treatment monitoring. Available data suggest that RISK6 demonstrates promising but context-dependent diagnostic performance and represents a versatile host-response biomarker across multiple clinical applications. However, variability across populations and the limited evidence in multidrug-resistant TB remain important constraints. In practice, RISK6 is unlikely to function optimally as a standalone biomarker. Its clinical value appears greater when interpreted within integrated frameworks that combine transcriptomic, microbiological, and clinical data. Further validation in diverse populations and real-world settings will be essential to support meaningful clinical implementation.

## 1. Introduction

Tuberculosis is one of the leading causes of death by a single infectious agent worldwide, resulting in approximately 10 million new cases and 1.3 million deaths per year [1]. Although advanced diagnostic technologies exist, current strategies rely on sputum-based testing methods such as microscopy and molecular assays [2,3]. These tools have significant limitations, mainly in patients with paucibacillary disease, extrapulmonary TB or if one is unable to produce sputum, and they provide little information regarding disease activity or response to treatment.

Moreover, immunological studies including interferon-γ release assays (IGRAs) cannot separate latent TB infection (LTBI) from active TB disease, and possess little predictive value for progression [2,3]. For that reason, there are also still key gaps in both early diagnosis and risk stratification and longitudinal monitoring—necessary tools for TB control in these very challenging settings.

To overcome these challenges, there is growing interest in host-derived biomarkers that describe the interaction between Mycobacterium TB and the host immune system. Whole-blood gene expression profiling has yielded host RNA transcriptomic signatures, which have shown great potential to capture dynamic immune responses across the disease spectrum of TB [4,5]. Such signatures offer a systems-level view of disease behavior and offer potential utility for identifying active TB, predicting progression from latent infection and monitoring treatment response [6,7,8].

Parsimonious gene signatures, among the number of transcriptomic models, have attracted particular attention owing to their clinical translation potential. The RISK6 signature, a six-gene ratio-based transcriptomic model, is one of the most thoroughly validated host biomarkers in TB. In contrast to previous multi-gene signatures, RISK6 adopts a pair-ratio strategy that increases analytical robustness and lowers technical variability and supports deployment using quantitative PCR-based platforms [9].

Accumulating evidence shows that RISK6 can effectively differentiate between active TB and latent infection and healthy controls, as well as determine short-term progression to disease [10,11,12]. In addition, RISK6 scores are also shown to decrease during effective anti-TB therapy which reflects dynamic alterations of disease activity thus justifying RISK6 as a treatment monitoring biomarker [9,13]. These phenotypes, together with the robust response profiles, provide an attractive basis for using RISK6 as a biomarker with multiple clinical applications for the continuum of TB care.

But even with such promising data, several key points remain. Gene function of transcriptomic signatures differ by population and geographical location and prediction is degraded with time, and its accuracy drops over long time horizons [11,12,14]. Also, there is little evidence on drug-resistant TB populations which is a clear gap considering growing global prevalence of MDR-TB and need of better techniques for treatment and monitoring.

In context of this literature, a systematic and translational synthesis of the available evidence is necessary to progress beyond a descriptive summary to a clinic-specific structure. This review presents an integrated evaluation of host RNA transcriptomic biomarkers in the context of TB, particularly the RISK6 signature as a novel translational platform for host RNA transcriptomes. We look at its biological basis, its clinical applications through diagnosis, prognosis, and treatment monitoring, and its place in drug-resistant TB, and highlight major challenges and future lines of clinical action.

Importantly, although multiple transcriptomic signatures have been proposed, their clinical translation has been inconsistent across settings. This highlights the need for approaches that balance biological performance with scalability and clinical feasibility.

## 2. Materials and Methods

### 2.1. Study Design

This study was conducted as a structured narrative review with a clinically oriented synthesis of host RNA transcriptomic biomarkers in TB, with a particular focus on the RISK6 signature. The review was informed by a systematic and comprehensive literature search to ensure transparency and reproducibility, while maintaining a qualitative and translational perspective.

A two-stage selection strategy was applied to ensure both broad coverage of the transcriptomic biomarker literature and in-depth evaluation of clinically relevant RISK6 studies. This approach allows integration of biological, clinical, and translational insights beyond purely quantitative synthesis.

### 2.2. Literature Search Strategy

A comprehensive and structured literature search was conducted to identify studies evaluating host RNA transcriptomic biomarkers in TB. Electronic databases, including PubMed, Scopus, and Web of Science, were searched for articles published up to December 2025.

The search strategy combined Medical Subject Headings (MeSH) and free-text terms using Boolean operators. Key search terms included “tuberculosis”, “RISK6”, “host RNA”, “transcriptomic signature”, “gene expression”, “biomarker”, “treatment monitoring”, and “disease progression”.

In addition, reference lists of relevant articles were manually screened to identify further eligible studies. The search was designed to capture both foundational studies on transcriptomic biomarkers and key studies specifically evaluating the RISK6 signature.

### 2.3. Eligibility Criteria

Studies were included if they met the following criteria:

1. Evaluated host RNA transcriptomic signatures in TB
2. Conducted in human populations
3. Reported clinically relevant applications, including diagnosis, prediction of disease progression, or treatment monitoring

Studies were excluded if they met any of the following criteria:

1. Did not involve transcriptomic biomarkers
2. Focused exclusively on non-human models
3. Were non-original publications without primary data (e.g., editorials, commentaries, or opinion articles)
4. Were not available in English

These criteria were applied to ensure inclusion of studies with direct clinical relevance and sufficient methodological detail for qualitative synthesis.

### 2.4. Study Selection

Study selection was conducted using a structured two-stage approach.

In the first stage, all identified records underwent title and abstract screening to identify studies evaluating host RNA transcriptomic signatures in TB. Full-text assessment was subsequently performed based on predefined eligibility criteria.

In the second stage, a focused selection was undertaken to identify studies specifically evaluating the RISK6 transcriptomic signature with clearly defined clinical applications, including diagnosis, prognosis, or treatment monitoring.

A total of 866 records were initially identified. After removal of duplicates and screening, 67 studies met the inclusion criteria for host RNA transcriptomic signatures and formed the broader evidence base. From these, 6 key studies specifically evaluating RISK6 were selected for in-depth qualitative synthesis. The broader transcriptomic evidence base was used to provide contextual background for the focused analysis of RISK6. This approach allowed integration of a broad transcriptomic evidence base with a focused evaluation of RISK6 for clinically relevant interpretation.

**Figure 1:**
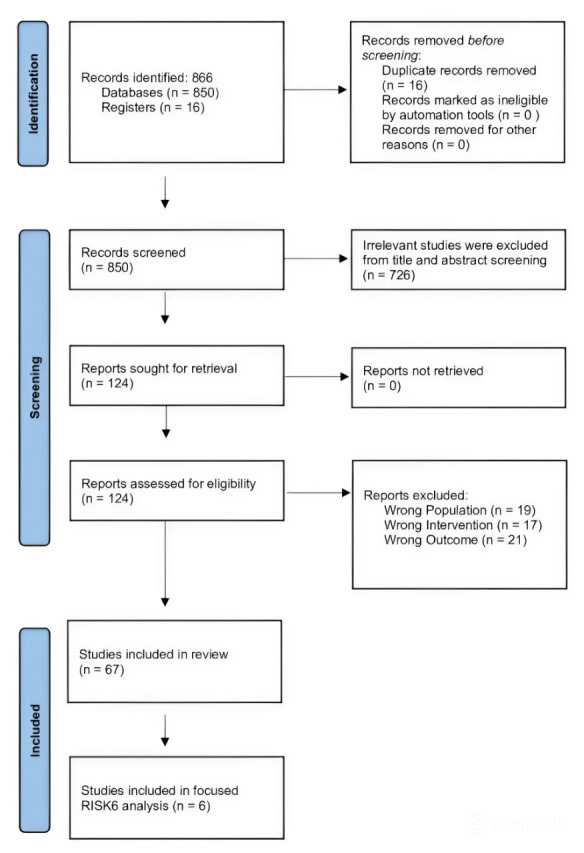
Overview of study inclusion for the focused analysis of RISK6 transcriptomic studies. Studies evaluating the RISK6 transcriptomic signature were identified from the broader literature on host RNA biomarkers in TB. A focused subset of studies with clearly defined clinical applications was selected for in-depth qualitative synthesis.

An overview of the study inclusion process for the focused analysis of RISK6 is presented in Figure 1.

This structured approach enabled integration of a broad transcriptomic evidence base with a focused evaluation of clinically relevant RISK6 studies.

### 2.5. Data Extraction and Synthesis

Data were extracted using a predefined framework, including study design and population characteristics, type of transcriptomic signature, clinical application (diagnostic, prognostic, or treatment monitoring), key findings, performance metrics (e.g., area under the curve (AUC), sensitivity, and specificity), and reported limitations.

Given the heterogeneity of study designs, populations, clinical objectives, and analytical approaches, a structured narrative synthesis was performed. Substantial heterogeneity across included studies limits direct comparability of reported performance metrics and precludes robust quantitative synthesis or formal meta-analysis.

The synthesis therefore emphasized clinical applicability, biological relevance, and translational potential of the RISK6 signature across different contexts. This approach is consistent with established methodological guidance for narrative synthesis in the presence of substantial clinical and methodological heterogeneity. This approach allowed for a clinically oriented interpretation of evidence while accounting for variability across study settings.

### 2.6. Analytical Framework

To enhance clinical interpretability and ensure a structured synthesis of findings, the included studies were categorized into three principal domains: (1) diagnostic application, (2) prognostic application (prediction of disease progression), and (3) treatment monitoring.

In addition, a translational framework was applied to evaluate the potential integration of RISK6 into clinical practice, including its relevance in drug-resistant TB and its use in conjunction with microbiological and clinical parameters. This framework guided the organization and interpretation of evidence throughout the review.

### 2.7. Methodological Quality Assessment

The methodological quality of included studies was assessed using established tools based on the primary clinical application of each study. QUADAS-2 was applied to studies evaluating diagnostic or triage performance, while QUIPS was used for studies focusing on prognostic or treatment-response outcomes.

For studies reporting multiple clinical applications, risk-of-bias assessment was conducted separately for each application rather than assigning a single overall judgment per study.

**Figure 2:**
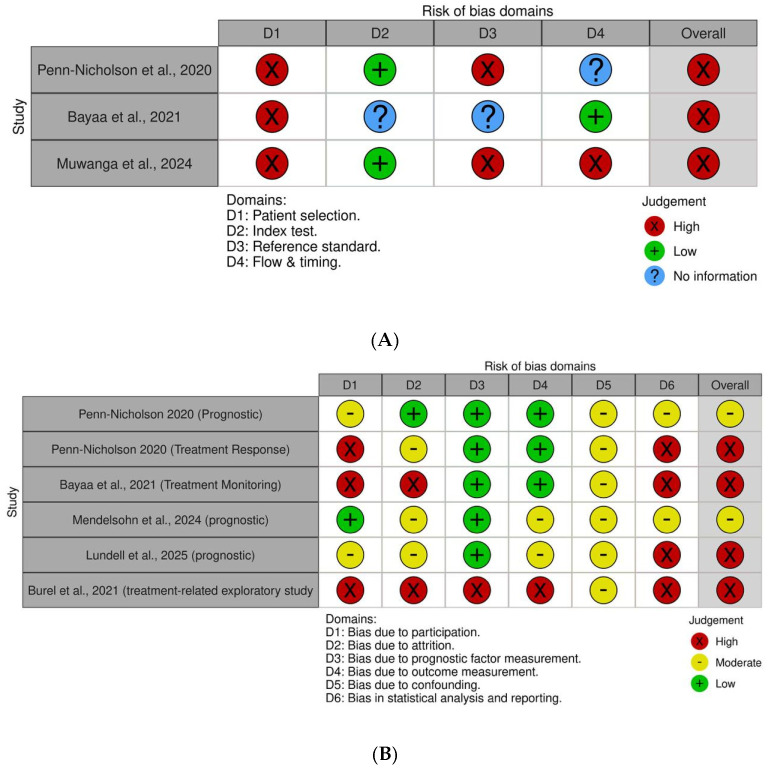
Risk-of-bias assessment of included studies using QUADAS-2 and QUIPS tools. (**A**) QUADAS-2 assessment of studies evaluating diagnostic performance, including domains of patient selection, index test, reference standard, and flow and timing. (**B**) QUIPS assessment of prognostic and treatment-response analyses, including domains of study participation, attrition, prognostic factor measurement, outcome measurement, confounding, and statistical analysis. Traffic light colors indicate risk of bias: green (low risk), yellow (moderate risk), red (high risk), and blue (unclear risk) [9,10,11,12,15,16].

Domain-level assessments were summarized using traffic plots, as presented in Figure 2. This approach enabled a more nuanced evaluation of study quality across different clinical use cases of RISK6.

## 3. Results

### 3.1. Results of Study Selection

A total of 866 records were initially identified through the literature search. After removal of duplicates and initial screening of titles and abstracts, 124 articles underwent full-text assessment. Of these, 67 studies met the inclusion criteria for host RNA transcriptomic signatures and formed the broader evidence base.

Given the focused objective of this review, a second-stage selection was conducted to identify studies specifically evaluating the RISK6 transcriptomic signature. A total of 6 studies met these criteria and were included in the final qualitative synthesis.

An overview of the study inclusion for the focused analysis of RISK6 is presented in Figure 1. The key characteristics and findings of the included RISK6 studies are summarized in Table 1. This structured approach allowed integration of a broad transcriptomic evidence base with a focused evaluation of clinically relevant RISK6 studies.

### 3.2. Risk of Bias Assessment

Risk-of-bias assessment was performed using QUADAS-2 for diagnostic studies and QUIPS for prognostic and treatment-response analyses. Domain-level judgments across included studies are summarized in Figure 2.

Diagnostic studies demonstrated a predominance of high risk of bias, particularly in the domains of patient selection and reference standard. These limitations reflect non-random sampling strategies and variability in comparator definitions across studies. In contrast, the index test domain generally showed lower risk, indicating consistent application of the RISK6 assay.

For prognostic and treatment-response analyses, QUIPS assessment revealed more heterogeneous study quality. Cohort-based studies showed relatively lower risk in outcome measurement and prognostic factor assessment, whereas concerns remained regarding attrition, confounding, and statistical analysis. Exploratory and treatment-monitoring studies exhibited higher risk of bias, particularly in participation and analysis domains.

Overall, these findings highlight that while RISK6 studies provide valuable insights, methodological limitations should be considered when interpreting reported performance across different clinical applications. These findings underscore the importance of cautious interpretation and context-specific validation of RISK6 across diverse clinical settings.

Overall, diagnostic studies demonstrated a predominance of high risk of bias, particularly in the domains of patient selection and reference standard, reflecting non-random sampling strategies and variability in comparator definitions. In contrast, the index test domain generally showed lower risk, indicating consistent implementation of the RISK6 assay.

For prognostic and treatment-response analyses, methodological quality was more heterogeneous. Cohort-based studies showed relatively lower risk in outcome measurement and prognostic factor domains, whereas concerns remained regarding attrition, confounding, and statistical analysis. Exploratory and treatment-monitoring studies exhibited higher risk of bias across multiple domains. These findings highlight the importance of cautious interpretation of reported performance metrics across different clinical applications.

### 3.3. Biological Basis of RISK6

The RISK6 transcriptomic signature reflects host immune responses to Mycobacterium tuberculosis (M. Tb), primarily driven by interferon-mediated pathways associated with TB pathogenesis [4,5]. Both type I and type II interferon signaling are strongly linked to active disease and are characterized by the upregulation of genes involved in innate immune activation, antimicrobial defense, and inflammatory responses [4]. These transcriptomic changes represent a systemic host response that extends beyond localized pulmonary infection.

RISK6 comprises six genes arranged in paired ratios, including three upregulated genes (GBP2, FCGR1B, and SERPING1) and three downregulated genes (TUBGCP6, TRMT2A, and SDR39U1) [9]. The upregulated genes are predominantly associated with interferon-inducible pathways and immune activation, whereas the downregulated genes are related to cellular homeostasis and metabolic regulation. This bidirectional expression pattern enhances the discriminatory capacity of the signature and provides a biologically meaningful representation of disease activity.

Importantly, alterations in gene expression can be detected prior to clinically apparent disease, consistent with the concept of incipient or subclinical TB [6,7,17,18]. These early changes reflect progressive immune activation, particularly involving interferon signaling, as the disease transitions from latent infection to active TB. This temporal dynamic underpins the ability of transcriptomic biomarkers such as RISK6 to predict short-term disease progression.

Methodologically, the ratio-based design of RISK6 offers advantages over approaches relying on absolute gene expression. By normalizing gene expression within paired ratios, this approach reduces technical variability across platforms and sample conditions, thereby improving analytical robustness and facilitating clinical translation using PCR-based assays [9,19,20].

Beyond its mechanistic basis, RISK6 captures the dynamic host immune response across the TB disease spectrum. Higher RISK6 scores reflect increased immune activation during progression from latent infection to active disease, whereas declining scores during treatment are associated with resolution of inflammatory responses [9]. This dynamic behavior supports its utility as a biomarker of disease activity rather than a static diagnostic measure.

Overall, the biological basis of RISK6 is grounded in well-characterized immunopathological mechanisms of TB, supporting its role as a dynamic indicator of host response across different stages of infection. This mechanistic foundation provides a strong rationale for its clinical and translational applications.

**Figure 3:**
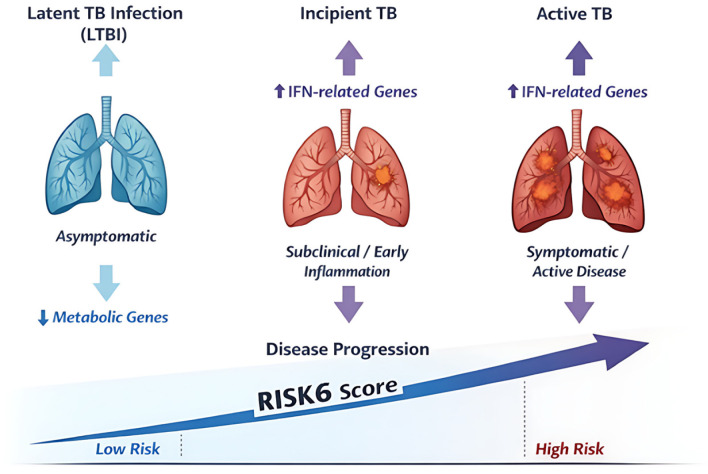
Biological basis and disease progression reflected by the RISK6 transcriptomic signature. The transition from LTBI to incipient and active TB is characterized by increased expression of interferon (IFN)-related genes and decreased expression of metabolic genes. These changes are associated with progressive disease activity and are reflected by an increasing RISK6 score, corresponding to a shift from low to high risk across the TB disease spectrum.

The biological basis of RISK6 and its relationship to disease progression across the TB spectrum are illustrated in Figure 3. These biological characteristics form the basis for the clinical utility of RISK6 across diagnosis, risk stratification, and treatment monitoring.

While the biological basis of RISK6 provides important mechanistic insights, its clinical value is best demonstrated through evidence from studies evaluating its performance across different applications. (Explanatory information has been added in the figure legend indicating that upward arrows (↑) denote increased/upregulated expression or rising disease activity, downward arrows (↓) denote decreased/downregulated expression, and the large directional arrow represents progression across the TB disease spectrum with increasing RISK6 score and risk.)

### 3.4. Evidence from RISK6 Studies

While the biological basis of RISK6 provides important mechanistic insights into host immune responses in TB, its clinical value is best demonstrated through evidence from studies evaluating its performance across different applications. The key characteristics and findings of these studies are summarized in Table 1, including study setting, HIV status, and sample size to highlight heterogeneity across included populations.

Collectively, these findings support the potential clinical utility of RISK6 across multiple applications, although interpretation should consider variability across populations and study settings. These observations provide a foundation for understanding the translational potential of RISK6 in clinical practice.

### 3.5. Translational Framework of RISK6

**Figure 4:**
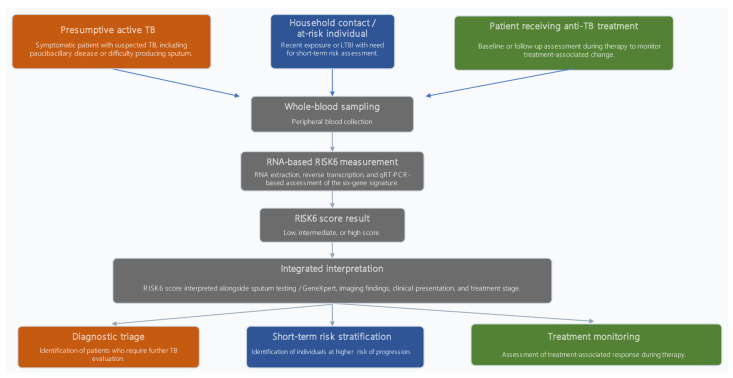
Proposed translational workflow for the clinical application of the RISK6 transcriptomic signature in TB. This framework highlights the role of RISK6 as an adjunct biomarker that complements microbiological and clinical assessment rather than replacing them. It also emphasizes its multi-functional utility across diagnostic triage, short-term risk stratification, and treatment monitoring within an integrated clinical approach.

While the available studies provide important evidence on the performance of RISK6, these findings can be more effectively interpreted within a translational framework that links biological mechanisms to clinical application. To address this, a conceptual model is proposed to illustrate the integration of RISK6 into clinical workflows across the TB care continuum (Figure 4). This model provides a unifying conceptual framework for integrating RISK6 into clinical decision-making.

This proposed workflow represents a unifying conceptual framework for integrating RISK6 into clinical decision-making across the TB care continuum. It synthesizes biological rationale, clinical evidence, and practical application into a single model that links host-response biomarkers with real-world clinical pathways. Taken together, this framework highlights the translational potential of RISK6 within integrated TB care pathways.

## 4. Discussion

### 4.1. Clinical Applications of RISK6

Beyond summarizing existing evidence, this review proposes a unified conceptual framework (Figure 4) to guide the clinical integration of RISK6. This framework links patient context, biomarker measurement, and multi-modal data integration to support decision-making across diagnosis, risk stratification, and treatment monitoring.

The RISK6 transcriptomic signature demonstrates clinically relevant utility across several key domains of TB management, including diagnosis, prediction of disease progression, and treatment monitoring. Unlike conventional pathogen-based diagnostics, which primarily reflect bacillary detection, RISK6 captures dynamic host immune responses associated with disease activity. This feature makes RISK6 particularly attractive as a clinically informative biomarker, as it provides complementary insight into both biological activity and treatment response rather than functioning solely as a static diagnostic readout. Collectively, the available evidence suggests that the greatest value of RISK6 lies not in a single isolated application, but in its potential to serve as a multi-purpose biomarker platform across the continuum of TB care.

#### 4.1.1. Diagnostic Application

RISK6 has demonstrated generally strong diagnostic performance in distinguishing active TB from latent infection and healthy individuals across multiple cohorts, with reported area under the curve (AUC) values approaching 0.94 and favorable sensitivity and specificity profiles [10,15]. However, performance variability across populations and clinical settings should be considered when interpreting these findings.

From a clinical perspective, RISK6 is particularly relevant in settings where conventional microbiological diagnostics are limited, such as in patients with paucibacillary disease, extrapulmonary TB, or inability to produce sputum. In contrast to pathogen-based assays, host transcriptomic biomarkers reflect the overall immune response to infection, thereby providing complementary information to microbiological testing [2,3].

However, this host-response-based approach is inherently associated with reduced specificity, as interferon-driven gene expression may also be observed in other infectious or inflammatory conditions [4,5]. This limitation underscores the importance of interpreting RISK6 within a broader clinical and microbiological context.

Taken together, the available evidence supports the role of RISK6 as a potential triage tool within integrated diagnostic frameworks, rather than as a standalone replacement for established microbiological methods.

#### 4.1.2. Prognostic Application (Prediction of Disease Progression)

One of the most clinically significant applications of RISK6 is its ability to predict progression from latent TB infection to active disease. Prospective cohort studies have demonstrated that RISK6 performs best in identifying individuals at imminent risk of developing active TB, particularly within a short-term window of approximately 6–9 months prior to clinical diagnosis [8,11].

This temporal performance aligns with the concept of incipient TB, in which early immune activation precedes overt clinical disease. In this context, elevated RISK6 scores reflect underlying interferon-driven immune responses associated with subclinical disease progression, supporting its role as an early warning biomarker.

Importantly, variability in prognostic performance across populations remains a critical consideration. Differences in geographic setting, host genetic background, and epidemiological context may influence the predictive accuracy of transcriptomic signatures. In particular, HIV co-infection has been shown to significantly alter host immune responses and gene expression profiles, potentially affecting the performance of RISK6 and other interferon-driven signatures [5,21].

These findings underscore the need for population-specific validation and careful interpretation of prognostic results in diverse clinical settings

However, the predictive performance of RISK6 declines over longer follow-up intervals, limiting its utility for long-term risk stratification [11,19,20]. This suggests that RISK6 is most effective when applied within defined screening intervals rather than as a single baseline assessment.

From a clinical standpoint, these findings position RISK6 as a tool for short-term risk enrichment, enabling identification of individuals who may benefit from targeted preventive interventions. Nevertheless, its optimal use requires integration into longitudinal or repeated testing strategies to maximize predictive accuracy.

Overall, RISK6 should be interpreted as a dynamic prognostic biomarker for incipient TB, rather than a static predictor of long-term disease risk.

#### 4.1.3. Treatment Monitoring

RISK6 has demonstrated substantial potential as a biomarker for monitoring treatment response in TB. Multiple studies have shown that RISK6 scores decline significantly during effective anti-TB therapy, reflecting reductions in disease activity and normalization of host immune responses [9,15,17,22].

This dynamic behavior distinguishes RISK6 from conventional microbiological markers, as transcriptomic changes may occur earlier than sputum culture conversion or smear negativity. As such, RISK6 has the potential to serve as an early indicator of treatment efficacy, providing clinically actionable information during the initial phases of therapy.

It is important to distinguish between validated findings and emerging evidence in this context. The consistent decline of RISK6 scores during effective therapy has been demonstrated across multiple cohorts, representing a relatively well-established observation [9,15,22]. In contrast, its role as an early surrogate marker for treatment outcomes or as a predictor of treatment failure remains an area of emerging evidence that requires further prospective validation.

From a practical perspective, this application is particularly valuable in patients with smear-negative or extrapulmonary TB, where microbiological monitoring is limited or delayed. In such settings, host-response biomarkers may provide an alternative means of assessing treatment response.

However, several challenges remain. The absence of standardized thresholds for defining treatment response limits clinical applicability, and variability across populations may affect longitudinal interpretation. In addition, current evidence remains limited in MDR-TB, where treatment response assessment is most critical.

Taken together, RISK6 represents a promising dynamic biomarker for treatment monitoring, with particular potential for early response assessment. Its clinical utility is likely to be maximized when integrated with microbiological and clinical parameters rather than used as a standalone tool.

#### 4.1.4. Integration into Clinical Practice

Based on the available evidence, RISK6 shows strong potential for integration into clinical workflows as part of a multi-modal diagnostic and monitoring strategy. Rather than replacing existing microbiological methods, RISK6 is best positioned as a complementary biomarker that enhances clinical decision-making by providing insight into host immune activity.

One of the most promising applications is the integration of RISK6 with established microbiological parameters, such as time to positivity (TTP) and sputum culture conversion, to improve assessment of disease activity and treatment response [22]. This combined approach may enable a more comprehensive evaluation of both bacillary burden and host response, particularly in complex clinical scenarios.

In addition, the use of RISK6 may support risk stratification and individualized patient management, aligning with the broader shift toward precision medicine in TB care. By identifying patients at higher risk of disease progression or suboptimal treatment response, RISK6 has the potential to inform more tailored therapeutic and monitoring strategies. In this context, RISK6 may serve as a decision-support biomarker within integrated clinical pathways, informing key steps such as triage testing, initiation of therapy, and longitudinal monitoring, particularly when combined with microbiological and clinical parameters.

However, successful clinical implementation requires several prerequisites, including assay standardization, definition of clinically meaningful thresholds, and validation across diverse populations. Furthermore, integration into routine care will depend on the development of scalable, cost-effective platforms that are feasible in resource-limited settings.

Overall, RISK6 is unlikely to function as a standalone clinical tool but may provide the greatest value when incorporated into integrated diagnostic and monitoring frameworks combining transcriptomic, microbiological, and clinical data.

#### 4.1.5. Relevance to Drug-Resistant TB

Despite the growing interest in host RNA transcriptomic biomarkers, evidence regarding the performance of RISK6 in drug-resistant TB remains limited. Most available studies have been conducted in drug-sensitive populations, leaving a significant gap in understanding its applicability in MDR-TB [14].

From a mechanistic perspective, it can be hypothesized that persistent or dysregulated interferon-driven immune activation may contribute to suboptimal treatment response in MDR-TB. In this context, sustained elevation of RISK6 scores during therapy could reflect ongoing host inflammatory activity despite microbiological treatment, potentially identifying patients at risk of delayed culture conversion or treatment failure. This hypothesis is biologically plausible given the central role of interferon signaling in TB pathogenesis and its association with disease activity.

This gap is particularly important given the clinical complexity of MDR-TB, which is characterized by prolonged treatment duration, higher rates of treatment failure, and limited options for microbiological monitoring. In this context, a host-response biomarker such as RISK6 may offer additional value by capturing dynamic changes in immune activity that are not fully reflected by conventional microbiological measures. If validated, this relationship would position RISK6 as a potential early indicator of treatment response in MDR-TB, complementing conventional microbiological markers such as sputum culture conversion and time to positivity, and enabling earlier identification of patients who may require treatment modification or intensified monitoring. This may enable earlier identification of patients at risk of suboptimal treatment response, thereby facilitating timely treatment modification or intensified clinical monitoring.

From a theoretical and translational perspective, RISK6 has the potential to contribute to early treatment response assessment and identification of patients at risk of poor outcomes or treatment failure. Such applications are especially relevant in MDR-TB, where timely evaluation of treatment efficacy remains a major clinical challenge.

However, the lack of robust validation studies in MDR-TB populations limits current clinical applicability. Differences in disease biology, treatment regimens, and host immune responses may influence transcriptomic profiles, necessitating dedicated studies in drug-resistant cohorts.

Overall, while RISK6 represents a promising candidate biomarker for MDR-TB, its role remains exploratory and requires further validation before integration into clinical practice.

This integrated approach aligns with the shift toward precision TB medicine, where biomarker-guided strategies may enable earlier diagnosis, improved risk stratification, and more responsive treatment monitoring. The clinical applications of RISK6 across diagnosis, prognosis, and treatment monitoring, along with their strengths, limitations, and translational implications, are summarized in Table 2.

Emerging evidence further suggests that combining transcriptomic signatures may enhance predictive performance, indicating that RISK6 is likely to be most effective as part of integrated biomarker strategies rather than as a standalone tool [16].

#### 4.1.6. Translational Perspective

Taken together, the available evidence indicates that RISK6 functions as a multi-dimensional biomarker capable of capturing dynamic host immune responses across the TB disease spectrum. Its consistent performance across diagnostic, prognostic, and treatment monitoring applications highlights its potential as a unified platform rather than a single-purpose tool.

From a translational standpoint, the greatest strength of RISK6 lies in its parsimonious design and ratio-based structure, which enhance analytical robustness and facilitate implementation using simplified platforms such as quantitative PCR [9,19,20]. This makes RISK6 particularly attractive for deployment in resource-limited settings, where scalable and cost-effective diagnostic tools are critically needed.

However, current evidence suggests that the clinical value of RISK6 is maximized when interpreted within integrated frameworks that combine transcriptomic, microbiological, and clinical parameters [16]. Such an approach aligns with the evolving paradigm of precision TB medicine, in which biomarker-guided strategies may enable earlier diagnosis, improved risk stratification, and more responsive treatment monitoring.

Importantly, RISK6 should not be viewed as a replacement for existing diagnostic or monitoring tools, but rather as a complementary component within a broader clinical decision-making framework. Future research should therefore focus on standardization of assay platforms, validation across diverse populations, and integration into real-world clinical workflows.

Overall, RISK6 represents a promising translational biomarker platform with the potential to bridge key gaps in TB management, although further validation is required to support widespread clinical implementation.

This framework provides a conceptual basis for integrating RISK6 into real-world clinical decision pathways.

### 4.2. Limitations and Challenges

Despite the promising potential of RISK6 as a host transcriptomic biomarker, several important limitations must be considered before its translation into routine clinical practice. The marked heterogeneity across included studies limits direct comparability of reported performance metrics and constrains the ability to perform quantitative synthesis. The variability in study setting, HIV status, and cohort size further contributes to heterogeneity in reported performance metrics. These challenges span biological variability, methodological constraints, and implementation barriers, reflecting the inherent complexity of host-response-based biomarkers in TB. These observations underscore the importance of context-specific validation in diverse populations.

The key limitations are discussed in the following sections.

#### 4.2.1. Variability Across Populations

A major limitation of RISK6 is the variability in performance across different geographic and epidemiological settings. Multicohort studies have demonstrated that transcriptomic signatures, including RISK6, do not consistently meet target product profile benchmarks when applied across diverse populations [12,16].

This variability is likely driven by differences in host genetic background, environmental exposures, TB endemicity, and the presence of comorbid conditions. In particular, co-infections such as HIV may substantially alter immune responses and gene expression profiles, thereby affecting the diagnostic and prognostic performance of transcriptomic biomarkers [5,21].

These findings highlight the need for context-specific validation and potential recalibration of RISK6 before clinical implementation. Without such adaptation, the generalizability of the signature across different populations remains limited.

#### 4.2.2. Limited Long-Term Predictive Performance

While RISK6 demonstrates strong performance in predicting short-term progression to active TB, its predictive accuracy declines over longer follow-up intervals. Several studies have shown that transcriptomic signatures perform optimally within a defined temporal window, typically within 6–12 months prior to disease onset, with reduced sensitivity and specificity beyond this period [8,11].

This temporal limitation suggests that RISK6 primarily captures biological processes associated with incipient or subclinical TB rather than long-term susceptibility to disease. As a result, its utility as a standalone tool for long-term risk stratification remains limited.

From a clinical perspective, this constraint implies that single baseline measurements may be insufficient for sustained risk prediction. Instead, repeated or interval-based testing strategies may be required to maintain predictive accuracy over time.

Overall, the time-dependent nature of RISK6 highlights the importance of aligning its clinical application with appropriate screening intervals, reinforcing its role as a dynamic biomarker for short-term risk assessment rather than a static predictor of long-term disease risk.

#### 4.2.3. Limited Specificity of Interferon-Driven Signatures

A fundamental limitation of RISK6 lies in its reliance on interferon-driven gene expression, which is not specific to TB. Interferon-inducible pathways are activated in a wide range of infectious and inflammatory conditions, including viral infections and autoimmune diseases, leading to overlapping transcriptomic profiles [4,5].

As a result, elevated RISK6 scores may not exclusively reflect TB-related immune responses, increasing the risk of false-positive interpretations in certain clinical contexts. This limitation is inherent to host-response-based biomarkers and represents a key challenge in distinguishing TB from other inflammatory states.

From a clinical perspective, this reduced specificity underscores the need to interpret RISK6 results in conjunction with microbiological findings and clinical assessment. Reliance on transcriptomic signatures alone may lead to diagnostic ambiguity, particularly in settings with high prevalence of co-infections or systemic inflammatory conditions.

Overall, the lack of disease-specific transcriptional signatures highlights the importance of integrated diagnostic approaches, in which host transcriptomic biomarkers such as RISK6 are combined with pathogen-based and clinical data to improve diagnostic accuracy.

#### 4.2.4. Lack of Standardized Thresholds

Another important limitation of RISK6 is the lack of standardized thresholds for clinical interpretation. Although multiple studies have demonstrated strong discriminatory performance, there is currently no universally accepted cut-off value to define diagnostic or prognostic categories [12,23].

This lack of standardization limits comparability across studies and complicates the translation of RISK6 into clinical decision-making. Variations in study populations, assay platforms, and analytical methods may lead to differences in optimal threshold values, further contributing to heterogeneity in reported performance.

From a clinical perspective, the absence of defined thresholds poses a significant barrier to implementation, as clinicians require clear and actionable criteria to guide diagnosis, risk stratification, and treatment monitoring. Without standardized cut-offs, interpretation of RISK6 results remains context-dependent and may reduce confidence in its clinical use.

Overall, establishing robust and reproducible thresholds through large-scale validation studies is essential for the integration of RISK6 into routine clinical practice.

#### 4.2.5. Limited Evidence in Drug-Resistant TB

A critical limitation of the current evidence base is the limited availability of studies evaluating RISK6 in drug-resistant TB. Most published data have been derived from drug-sensitive TB populations, resulting in a significant gap in understanding the performance of RISK6 in MDR-TB [14].

This limitation is particularly important given the distinct clinical and biological characteristics of MDR-TB, including prolonged disease course, more complex treatment regimens, and higher rates of treatment failure. These factors may influence host immune responses and, consequently, transcriptomic signatures.

From a clinical perspective, the lack of data in MDR-TB restricts the generalizability of RISK6, especially in settings where drug resistance is prevalent. This represents a major barrier to implementation, as biomarkers intended for clinical use must demonstrate consistent performance across different disease subtypes.

Overall, further studies specifically designed to evaluate RISK6 in MDR-TB populations are essential to determine its role in treatment monitoring, prognosis, and clinical decision-making in this high-risk group.

#### 4.2.6. Need for Multi-Biomarker Approaches

An additional limitation of RISK6 lies in its use as a single biomarker to represent a highly complex and heterogeneous disease process. Tuberculosis pathogenesis involves dynamic interactions between host immunity, pathogen burden, and environmental factors, which may not be fully captured by a single transcriptomic signature.

Emerging evidence suggests that combining multiple biomarkers may improve diagnostic and prognostic performance compared to individual signatures [16,24,25]. Integrating transcriptomic data with microbiological, clinical, and radiological parameters may provide a more comprehensive assessment of disease activity and treatment response.

From a translational perspective, this supports a shift toward multi-biomarker frameworks, in which RISK6 functions as one component within a broader diagnostic and monitoring strategy rather than as a standalone tool. Such integrated approaches are more likely to improve accuracy, robustness, and clinical applicability across diverse patient populations.

Overall, the need for multi-biomarker approaches reflects the inherent complexity of TB and highlights the importance of combining complementary data sources to optimize clinical decision-making.

#### 4.2.7. Implementation Challenges

Beyond biological and methodological limitations, several practical challenges must be considered for the implementation of RISK6 in routine clinical settings. These include the cost of molecular testing, the requirement for laboratory infrastructure, and the need for trained personnel, particularly in resource-limited settings where the burden of TB is highest [2,3].

In addition, variability in assay platforms and lack of standardized protocols may affect reproducibility and comparability across different laboratories. Ensuring consistent sample collection, processing, and analysis is essential for reliable application of transcriptomic biomarkers in clinical practice.

From a health systems perspective, integration of RISK6 into existing diagnostic workflows presents further challenges. Adoption will depend not only on analytical performance but also on cost-effectiveness, accessibility, and compatibility with current clinical pathways.

Overall, addressing these implementation barriers will be critical to translating the potential of RISK6 into real-world clinical impact, particularly in high-burden and resource-constrained settings.

#### 4.2.8. Translational Perspective on Limitations

Taken together, these limitations highlight the inherent complexity of translating host transcriptomic biomarkers such as RISK6 into clinical practice. While individual challenges—ranging from biological variability and limited specificity to lack of standardization and implementation barriers—may appear distinct, they are closely interconnected and collectively influence real-world applicability.

From a translational perspective, these constraints underscore that RISK6 is unlikely to function effectively as a standalone biomarker. Instead, its clinical value is maximized when incorporated into integrated diagnostic and monitoring frameworks that combine transcriptomic, microbiological, and clinical data.

Furthermore, the need for repeated measurements, context-specific calibration, and standardized assay platforms reflects a broader shift toward dynamic and adaptive biomarker strategies in TB. This aligns with the evolving paradigm of precision medicine, where multi-dimensional data are used to guide individualized patient management.

Importantly, addressing these limitations does not diminish the value of RISK6, but rather defines the pathway for its optimal use. Future research should therefore prioritize multicohort validation, development of standardized thresholds, and integration into scalable clinical platforms to enable meaningful implementation.

Overall, the limitations of RISK6 should be viewed not as barriers, but as key considerations that inform its role as part of a broader, integrated biomarker ecosystem in TB care.

### 4.3. Comparison with Other Transcriptomic Signatures

The expanding landscape of host blood transcriptomic biomarkers in TB necessitates comparative evaluation of RISK6 against both earlier and more recent transcriptomic models. Although many signatures are derived from overlapping interferon-driven immune pathways, they differ substantially in gene number, intended clinical application, analytical complexity, and translational feasibility. Positioning RISK6 within this broader context is therefore essential to define its relative strengths, limitations, and potential clinical role.

#### 4.3.1. Comparison with Early Multi-Gene Signatures

Early transcriptomic signatures, including the ACS16 and ACS11 models, provided important proof of concept that whole-blood RNA profiles could predict progression to active TB before overt clinical disease [6,19,20]. These studies were foundational in establishing the biological relevance of host-response biomarkers and in demonstrating the prognostic potential of interferon-driven transcriptional signatures.

However, despite their predictive value, these earlier multi-gene models were relatively complex and therefore less suitable for routine clinical implementation. Larger gene sets increase analytical burden, require more extensive assay development, and limit scalability for near-patient testing.

In contrast, RISK6 was developed as a parsimonious six-gene signature using a pair-ratio approach designed to improve analytical robustness and reduce vulnerability to technical variation [9]. This reduction in gene number, together with its ratio-based structure, represents a substantial translational advantage by facilitating incorporation into simplified PCR-based platforms.

Taken together, the comparison with early multi-gene signatures suggests that the principal advantage of RISK6 lies not in replacing the biological insights generated by earlier models, but in translating those insights into a more practical and clinically deployable biomarker format.

#### 4.3.2. Comparison in Prognostic Performance

Among host RNA transcriptomic signatures, prognostic performance has been one of the most extensively studied applications, particularly in predicting progression from latent infection to active TB. Several early and contemporary signatures have demonstrated the ability to identify individuals at increased risk of developing active disease, especially within a defined short-term window prior to clinical diagnosis [6,11,19].

Comparative studies suggest that many transcriptomic models share similar predictive patterns, reflecting common underlying interferon-driven immune responses associated with incipient TB. However, differences in study design, population characteristics, and follow-up duration contribute to variability in reported performance across signatures.

In this context, RISK6 demonstrates prognostic performance comparable to larger and more complex models, particularly for short-term risk prediction within approximately 6–12 months before disease onset [8,11]. Importantly, its parsimonious structure enables this level of performance while maintaining greater analytical simplicity.

Nevertheless, as with other transcriptomic signatures, the predictive accuracy of RISK6 declines over longer time horizons, limiting its utility for long-term risk stratification. This limitation appears to be a common feature across prognostic models and reflects the dynamic nature of host immune activation during the transition from latent to active disease.

Overall, comparative evidence indicates that RISK6 performs similarly to other established transcriptomic signatures in prognostic applications, with the added advantage of a simplified and more clinically adaptable design.

#### 4.3.3. Comparison in Diagnostic Application

Host RNA transcriptomic signatures have been widely evaluated for their ability to distinguish active TB from latent infection and non-TB controls. Several models have demonstrated high diagnostic accuracy in controlled research settings, often achieving AUC values above 0.80 [10,11].

However, comparative analysis suggests that differences in diagnostic performance between individual signatures are often modest and highly dependent on study design, population characteristics, and reference standards. Larger gene signatures may show slightly higher accuracy in discovery cohorts, but these advantages are frequently reduced in validation studies and real-world settings.

In this context, RISK6 demonstrates diagnostic performance comparable to other established transcriptomic models, while offering important practical advantages related to its parsimonious design [9,15]. Its six-gene structure and ratio-based approach facilitate implementation using simplified platforms such as quantitative PCR, enhancing scalability and potential for clinical deployment.

Nevertheless, similar to other host-response biomarkers, RISK6 is limited by reduced specificity, as interferon-driven gene expression may overlap with other infectious and inflammatory conditions [4,5]. This limitation is not unique to RISK6 but reflects a broader challenge across transcriptomic diagnostic approaches.

Overall, comparative evidence indicates that RISK6 achieves diagnostic performance similar to more complex transcriptomic signatures, with the added benefit of improved translational feasibility, supporting its role as a practical component within integrated diagnostic frameworks.

#### 4.3.4. Comparison in Treatment Monitoring

The use of host RNA transcriptomic signatures for treatment monitoring has gained increasing attention, as these biomarkers capture dynamic changes in immune activity during therapy. Several transcriptomic models have demonstrated the ability to reflect treatment response, although the extent and consistency of these changes vary across signatures [9,17].

Compared with other transcriptomic approaches, RISK6 shows a consistent pattern of decline during effective anti-TB therapy, corresponding to reduced disease activity and normalization of host immune responses [9,15,22]. This dynamic behavior supports its potential role as a biomarker for monitoring treatment response.

Importantly, transcriptomic changes captured by RISK6 may occur earlier than conventional microbiological markers, such as sputum culture conversion, providing a potential advantage for early assessment of treatment efficacy. While similar trends have been reported in other signatures, the simplicity and reproducibility of RISK6 may enhance its practical utility in longitudinal monitoring.

However, comparative evidence remains limited, and variability in study design, sampling intervals, and patient populations complicates direct comparison between models. As such, while RISK6 appears promising for treatment monitoring, further head-to-head studies are needed to define its relative performance.

Overall, RISK6 demonstrates comparable or potentially advantageous performance in treatment monitoring relative to other transcriptomic signatures, particularly due to its consistent dynamic response and translational feasibility.

#### 4.3.5. Emerging Role of Composite Signatures

Recent developments in TB biomarker research have highlighted the potential advantages of composite or integrated biomarker approaches over single transcriptomic signatures. Given the complex and heterogeneous nature of TB, reliance on a single biomarker may be insufficient to fully capture disease dynamics.

Emerging evidence suggests that combining transcriptomic signatures with other biological and clinical parameters—such as microbiological data, radiological findings, and clinical scores—may improve diagnostic accuracy, prognostic performance, and treatment monitoring capabilities [16,24,25]. These integrated models aim to leverage complementary information from different domains to enhance overall predictive performance.

Within this evolving landscape, RISK6 may serve as a core component of composite biomarker frameworks rather than as a standalone tool. Its parsimonious structure, reproducibility, and demonstrated utility across multiple clinical applications make it well suited for integration into multi-modal diagnostic and monitoring strategies.

However, the development and validation of composite signatures remain in early stages, and challenges related to model complexity, standardization, and clinical implementation persist. Further studies are required to determine optimal combinations of biomarkers and to evaluate their performance in real-world settings.

Overall, the emergence of composite biomarker strategies represents a shift toward more comprehensive and precision-oriented approaches in TB care, in which RISK6 may play a central but complementary role.

#### 4.3.6. Overall Positioning of RISK6

Taken together, comparative evaluation of transcriptomic signatures indicates that RISK6 occupies a distinct position within the current biomarker landscape. While it does not consistently outperform all other models in every clinical application, it demonstrates a favorable balance between diagnostic performance, prognostic utility, and treatment monitoring capability.

A key strength of RISK6 lies in its parsimonious six-gene design and ratio-based structure, which enhance analytical robustness and facilitate translation into scalable platforms such as quantitative PCR [9,19]. In contrast to more complex multi-gene signatures, this simplified architecture improves feasibility for implementation in real-world clinical settings, particularly in resource-limited environments.

In addition, RISK6 demonstrates multi-purpose applicability across several stages of TB care, including diagnosis, prediction of short-term disease progression, and monitoring of treatment response. This versatility distinguishes it from many other transcriptomic models that are optimized for a single clinical function.

However, consistent with the broader limitations of host-response biomarkers, RISK6 should not be considered a standalone solution. Its optimal clinical value is likely achieved when integrated with microbiological and clinical parameters within multi-modal diagnostic and monitoring frameworks.

Overall, RISK6 represents a practical and translationally promising biomarker platform that bridges the gap between high-dimensional discovery models and clinically deployable tools, although further validation is required to support widespread implementation.

A comparative overview is presented in Table 3, with all statements supported by corresponding literature to ensure transparency and traceability.

Notably, the advantage of RISK6 lies less in consistently outperforming other signatures and more in achieving a pragmatic balance between performance, simplicity, and translational feasibility.

### 4.4. Future Perspectives and Relevance to Drug-Resistant TB

Future research on host transcriptomic biomarkers should move beyond proof-of-concept performance and focus on their integration into clinically meaningful and scalable frameworks. In this context, RISK6 is particularly relevant because its parsimonious design, dynamic behavior, and multi-purpose applicability position it as a promising candidate for translational development. At the same time, the greatest unmet need may lie in drug-resistant TB, where improved tools for risk stratification, treatment monitoring, and early identification of poor response are urgently required. Accordingly, future perspectives on RISK6 should be considered not only in terms of broader precision TB medicine, but also in relation to its potential contribution to MDR-TB care.

#### 4.4.1. Toward Precision TB Medicine

The future development of host transcriptomic biomarkers is closely aligned with the broader shift toward precision TB medicine. Rather than applying uniform diagnostic and monitoring strategies to all patients, precision approaches aim to tailor clinical decisions according to individual disease biology, risk of progression, and treatment response.

In this context, RISK6 represents a promising biomarker platform because it captures dynamic host immune activity across multiple stages of TB. Its demonstrated utility in diagnosis, short-term risk prediction, and treatment monitoring suggests that it may contribute to more individualized approaches to patient assessment and follow-up [9,11,16].

Importantly, the relevance of RISK6 to precision medicine lies not only in its performance characteristics, but also in its translational feasibility. Its parsimonious structure and ratio-based design make it more amenable to scalable implementation than larger and more complex transcriptomic models [9,19,20].

However, realization of this precision medicine framework will require more than biomarker discovery alone. Standardized assays, clinically validated thresholds, repeated-measurement strategies, and integration with existing clinical workflows will all be necessary to translate transcriptomic signatures into actionable tools for individualized TB care.

Overall, RISK6 aligns well with the evolving paradigm of precision TB medicine, in which biomarker-guided strategies may enable earlier diagnosis, more refined risk stratification, and more responsive treatment monitoring.

#### 4.4.2. Expanding the Role in MDR-TB

The potential role of RISK6 in MDR-TB represents a critical area for future research. Compared to drug-sensitive TB, MDR-TB is characterized by prolonged treatment duration, higher rates of treatment failure, and more complex clinical management, highlighting the need for improved biomarkers to guide patient care. To address these gaps, future research should adopt more rigorous and targeted study designs.

Future research should prioritize prospective longitudinal studies specifically designed to evaluate the relationship between RISK6 dynamics and treatment outcomes in MDR-TB. Such studies should incorporate serial blood sampling at predefined time points alongside microbiological assessments, including sputum culture conversion and time to positivity, to determine whether changes in RISK6 scores correlate with treatment response, failure, or relapse.

In addition, stratified analyses based on HIV status, disease severity, and treatment regimen may provide further insight into the variability of transcriptomic responses in MDR-TB populations. Integration of RISK6 into multi-modal biomarker models combining transcriptomic, microbiological, and clinical parameters should also be explored to improve predictive accuracy and clinical utility.

In this context, RISK6 may offer particular value as a dynamic host-response biomarker capable of capturing changes in disease activity and treatment response. Early identification of inadequate treatment response or risk of treatment failure is especially important in MDR-TB, where delays in clinical decision-making may lead to poorer outcomes.

Although current evidence remains limited, the biological basis of RISK6 suggests that it may be applicable across different forms of TB, including drug-resistant disease. However, differences in host immune response, treatment regimens, and disease progression patterns in MDR-TB may influence transcriptomic profiles and biomarker performance [14,27].

From a translational perspective, expanding the application of RISK6 to MDR-TB will require dedicated validation studies in drug-resistant cohorts, including longitudinal designs that assess its role in treatment monitoring and outcome prediction.

Overall, the extension of RISK6 into MDR-TB represents both a major opportunity and a critical research priority, with the potential to address important gaps in current TB management.

#### 4.4.3. Integration with Microbiological Markers

A key direction for future research is the integration of host transcriptomic biomarkers such as RISK6 with established microbiological parameters. While microbiological tests, including sputum culture and molecular assays, provide direct evidence of pathogen presence and bacillary burden, they offer limited insight into host immune activity and disease dynamics.

In contrast, transcriptomic signatures capture host-response patterns that reflect underlying inflammatory activity and treatment response. Combining these complementary data sources may provide a more comprehensive assessment of TB, integrating both pathogen burden and host immune status.

In particular, integration with microbiological markers such as TTP and culture conversion may enhance the evaluation of treatment response and disease activity [22]. Such combined approaches may enable earlier identification of treatment failure or delayed response, which is especially relevant in complex cases, including MDR-TB.

From a translational perspective, the integration of transcriptomic and microbiological data represents a critical step toward more precise and dynamic monitoring strategies. However, standardized frameworks for combining these biomarkers are still lacking, and further studies are needed to define optimal integration models.

Overall, combining RISK6 with microbiological markers may improve clinical decision-making by providing a more holistic view of TB disease processes.

#### 4.4.4. Toward Multi-Biomarker Models

Future advances in TB biomarker research are likely to move toward multi-biomarker models that integrate diverse sources of biological and clinical information. Given the complexity and heterogeneity of TB, reliance on a single biomarker is unlikely to provide sufficient accuracy across all clinical scenarios.

Multi-biomarker approaches aim to combine complementary data, including transcriptomic signatures, microbiological findings, radiological features, and clinical parameters, to improve overall diagnostic and prognostic performance [16,24,25]. Such models have the potential to capture different dimensions of disease biology, including pathogen burden, host immune response, and structural lung involvement.

Within this framework, RISK6 may serve as a central component due to its demonstrated utility across multiple clinical applications and its relative simplicity compared to larger transcriptomic signatures. Its integration into composite models may enhance predictive performance while maintaining feasibility for clinical implementation.

However, the development of multi-biomarker models introduces additional challenges, including increased model complexity, the need for standardized integration strategies, and validation across diverse populations. Ensuring that such models remain clinically practical and cost-effective will be essential for their successful translation.

Overall, the shift toward multi-biomarker approaches reflects an evolution from single-parameter diagnostics toward more comprehensive and individualized strategies for TB management.

#### 4.4.5. Translation into Clinical Practice

The successful translation of RISK6 into clinical practice will depend on multiple factors beyond analytical performance. While existing studies demonstrate promising diagnostic, prognostic, and treatment monitoring capabilities, several practical considerations must be addressed to enable real-world implementation.

A key requirement is the development of standardized and scalable assay platforms. The parsimonious six-gene structure of RISK6 makes it well suited for implementation using quantitative PCR-based systems, which are widely available and potentially adaptable for near-patient testing [9,19,20]. This represents a significant advantage over more complex transcriptomic signatures that require high-throughput sequencing or advanced computational analysis.

In addition, clear clinical use cases must be defined to guide integration into existing diagnostic and monitoring workflows. Potential applications include triage testing, short-term risk stratification, and early assessment of treatment response, particularly in settings where conventional microbiological tools are limited or delayed.

Implementation of RISK6 in clinical settings would require access to molecular diagnostic platforms, such as quantitative PCR systems, as well as standardized protocols for sample collection, processing, and analysis. In addition, trained laboratory personnel and quality control systems are essential to ensure reproducibility and reliability of results.

However, implementation will also require validation in diverse real-world populations, establishment of standardized thresholds, and demonstration of cost-effectiveness. At present, data on the cost-effectiveness of RISK6 are limited, and further studies evaluating its economic impact will be essential to support large-scale implementation. Health system factors, including infrastructure, training, and accessibility, will further influence adoption, particularly in high-burden, resource-limited settings [2,3].

While RISK6 shows strong translational potential, its clinical application remains investigational and requires further validation before routine implementation.

Overall, the successful translation of RISK6 into clinical practice will depend on coordinated efforts in assay development, validation, and integration into routine care pathways.

#### 4.4.6. Future Directions

Future research on RISK6 and other host transcriptomic biomarkers should prioritize large-scale, multicohort validation studies across diverse populations. Such efforts are essential to ensure generalizability and to address the variability in performance observed across different geographic and clinical settings.

In addition, the establishment of standardized assay platforms and clinically relevant thresholds will be critical to enable consistent interpretation and integration into clinical workflows. Longitudinal studies incorporating repeated measurements are also needed to better define the temporal dynamics of RISK6 and optimize its use in treatment monitoring and short-term risk prediction.

Another important direction is the expansion of research into underrepresented populations, particularly individuals with MDR-TB, HIV co-infection, and extrapulmonary disease. These groups represent key clinical scenarios in which improved biomarkers are urgently needed.

Furthermore, future studies should explore the integration of RISK6 into multi-biomarker frameworks that combine transcriptomic, microbiological, and clinical data. Advances in digital health and data integration platforms may facilitate the development of such models, enabling more precise and individualized approaches to TB management.

Future research should also prioritize standardization of assay platforms, validation across diverse populations, and prospective clinical trials to establish the clinical utility of RISK6 in real-world settings.

Overall, advancing RISK6 from a promising research tool to a clinically actionable biomarker will require coordinated efforts in validation, standardization, and integration, with a focus on real-world applicability and impact in high-burden settings.

## 5. Conclusions

Host RNA transcriptomic biomarkers represent a promising paradigm shift in TB management, offering the potential to complement conventional pathogen-based diagnostics by capturing dynamic host immune responses. Among these, RISK6 has emerged as a parsimonious and versatile host-response signature with demonstrated utility across diagnosis, short-term risk prediction, and treatment monitoring.

Comparative analysis indicates that while RISK6 does not consistently outperform all other transcriptomic signatures, it provides a favorable balance between performance, simplicity, and translational feasibility. Its ratio-based design and compatibility with scalable platforms such as quantitative PCR support its potential for real-world clinical implementation, particularly in resource-limited settings.

However, several challenges remain, including variability across populations, limited specificity of interferon-driven responses, lack of standardized thresholds, and insufficient validation in MDR-TB. These limitations highlight the need for further multicohort validation, assay standardization, and integration into multi-modal diagnostic and monitoring frameworks.

Looking forward, the greatest clinical value of RISK6 is likely to be realized not as a standalone biomarker, but as part of integrated strategies combining transcriptomic, microbiological, and clinical data. Such approaches align with the evolving paradigm of precision TB medicine, where biomarker-guided decision-making may enable earlier diagnosis, improved risk stratification, and more responsive treatment monitoring.

In conclusion, RISK6 represents a translationally promising biomarker platform that bridges the gap between high-dimensional discovery models and clinically deployable tools, although further research is required to support its widespread implementation in routine TB care.

Importantly, the proposed RISK6-guided framework provides a conceptual basis for translating transcriptomic biomarkers into clinically actionable decision pathways, supporting the integration of host-response signatures into precision TB care.

## Figures and Tables

**Table 1:** Key studies evaluating the RISK6 transcriptomic signature in TB and their clinical implications (n = 6).

Study	Population/ Setting	Setting	HIV Status	Sample Size	Design	Application	Key Findings	Performance	Limitations	Clinical Interpretation
Penn-Nicholson et al., 2020 [9]	Multicohort	Africa (South Africa)	Mixed	~600+	Prospective/validation	Diagnosis, prognosis, monitoring	Correlates with disease activity; declines during treatment	AUC > 0.85; dynamic change	Limited MDR-TB data; no standardized cut-off	Multi-purpose biomarker; promising for treatment monitoring
Bayaa et al., 2021 [10]	Multicountry cohorts	Africa, Asia, Eastern Europe	Mixed	~400–500	Validation	Diagnosis	Differentiates active TB vs LTBI/healthy	AUC ≈ 0.94; Se ~90.9%; Sp ~87.8%	Variability; overlap with inflammation	Potential triage tool (non-sputum-based)
Mendelsohn et al., 2024 [11]	Household contacts	Brazil	Mixed	~200–300	Prospective cohort	Prognosis	Strong short-term prediction (≤6–9 months)	Meets WHO TPP short-term	Weak ≥12 months	Best for short-term risk stratification (incipient TB)
Muwanga et al., 2024 [12]	Multicountry cohorts	Africa	Mixed	~300–400	Cross-cohort	Diagnosis, prognosis	Performance varies by geography	AUC ~0.75–0.85	Heterogeneity	Requires population-specific calibration
Burel et al., 2021 [15]	TB vs LTBI	Europe/mixed	Not specified	~100–200	Observational	Diagnosis	Distinguishes TB states (host response)	AUC ~0.85–0.90	Not RISK6-specific	Supports biological validity of host signatures
Lundell et al., 2025 [16]	Integrated cohorts	Brazil	Mixed	~200+	Modeling	Prognosis (composite)	Combined signatures ↑ accuracy	Se ~90%; Sp ~88%	Complexity	RISK6 may be stronger in composite panels

Collectively, the evidence highlights the multi-dimensional clinical utility of RISK6 across diagnosis, prognosis, and treatment monitoring. While strong diagnostic performance and short-term predictive accuracy are consistently observed, important limitations remain, including variability across populations, reduced long-term predictive performance, and limited validation in drug-resistant TB. These findings underscore the need for context-specific validation and support the integration of RISK6 within broader clinical and biomarker frameworks. (The arrows are intended to indicate directionality of change (↑ increased/upregulated; ↓ decreased/downregulated).

**Table 2:** Clinical Applications of RISK6 in TB.

Domain	Key Evidence	Strengths	Limitations	Clinical Role
Diagnosis	Bayaa et al. [10]; Muwanga et al. [12]; Burel et al. [15]	High diagnostic accuracy (AUC up to 0.94); non-sputum-based	Reduced specificity in inflammatory conditions; variability across populations	Potential triage tool, especially in paucibacillary TB
Prognosis (Disease Progression)	Penn-Nicholson et al. [9]; Mendelsohn et al. [11]	Strong short-term prediction (≤6–9 months); aligns with incipient TB concept	Declining performance over longer intervals	Useful for identifying high-risk individuals for targeted prevention
Treatment Monitoring	Penn-Nicholson et al. [9]; Muwanga et al. [12]	Dynamic decline during therapy; reflects disease activity	Lack of standardized thresholds; limited MDR-TB data	Potential early marker of treatment response and failure
Drug-Resistant TB (Emerging)	Limited evidence [14]	Theoretical relevance for response monitoring	Insufficient validation in MDR-TB populations	Promising but requires further validation
Integrated/Composite Biomarker Models	Lundell et al. [16]	Improved predictive accuracy when combined with other signatures	Increased complexity; reduced scalability	RISK6 may be most effective as part of multi-biomarker strategies

Overall, these findings highlight the role of RISK6 as a multi-dimensional biomarker platform with its greatest utility in short-term risk prediction and treatment monitoring, while emphasizing the importance of integration with clinical and microbiological parameters.

**Table 3:** Comparison of RISK6 with other blood transcriptomic signatures for TB.

Signature	No. of Genes	Primary Application	Performance Characteristics	Strengths	Limitations	Clinical Positioning
RISK6	6	Diagnosis, prognosis, monitoring	AUC up to 0.90–0.94; strong short-term prediction; dynamic treatment response [9,10,19,20]	Parsimonious; ratio-based; multi-functional; PCR-compatible [9,19]	Reduced long-term prediction; variability across populations; lack of standard cut-offs [14,22]	Most clinically versatile; suitable for integrated diagnostic and monitoring strategies [9,10,20]
ACS16/RISK16	16	Prognosis	Strong prediction of progression to active TB [6,8,17]	Well-characterized IFN-driven signature [4,6]	Large gene set; limited scalability [8,19]	Useful for research and risk prediction; less practical for routine use [6,8]
ACS11/RISK11	11	Prognosis	Moderate predictive performance [8,26]	Reduced gene set compared with ACS16 [8]	Limited long-term performance; reduced specificity [26]	Potential for targeted risk stratification with further validation [8,26]
PREDICT29	29	Prognosis	High performance in selected cohorts [8]	Broad biological coverage [8]	Complex; limited generalizability [8]	Promising but not yet clinically scalable [8]
Composite/Integrated Models	Variable	Prognosis, risk stratification	Improved sensitivity and specificity (~85–90% in modelling-based studies) [8,16,19]	Highest predictive accuracy; adaptable [16,19]	High complexity; cost; standardization challenges [16,22]	Likely future direction; RISK6 may serve as a component of integrated models [16,19]

Overall, while more complex signatures may offer higher predictive accuracy, RISK6 provides a favorable balance between performance, scalability, and clinical applicability, supporting its role within integrated biomarker strategies.

## Data Availability

No new data were created or analyzed in this study. Data sharing is not applicable to this article.

## Abbreviations

The following abbreviations are used in this manuscript:

TB	Tuberculosis
MDR-TB	Multidrug-Resistant Tuberculosis
LTBI	Latent Tuberculosis Infection
RISK6	Six-gene transcriptomic signature for tuberculosis risk, diagnosis, and treatment monitoring
RNA	Ribonucleic Acid
PCR	Polymerase Chain Reaction
IFN	Interferon
IGRA	Interferon-Gamma Release Assay
AUC	Area Under the Curve
TTP	Time to Positivity
WHO	World Health Organization
TPP	Target Product Profile
